# Deciphering the Controversial Role of TP53 Inducible Glycolysis and Apoptosis Regulator (TIGAR) in Cancer Metabolism as a Potential Therapeutic Strategy

**DOI:** 10.3390/cells14080598

**Published:** 2025-04-15

**Authors:** Fatima I. AlMaazmi, Lara J. Bou Malhab, Leen ElDohaji, Maha Saber-Ayad

**Affiliations:** 1 College of Medicine, University of Sharjah, Sharjah 27272, United Arab Emirates; u21106126@sharjah.ac.ae (F.I.A.); u24104154@sharjah.ac.ae (L.E.); 2 Research Institute for Medical and Health Sciences, University of Sharjah, Sharjah 27272, United Arab Emirates; lbonmalhab@sharjah.ac.ae; 3 Immunology and NAT, Dubai Blood Donation Center, Dubai Health, Dubai P.O. Box 505055, United Arab Emirates; 4 Faculty of Medicine, Cairo University, Cairo 11562, Egypt

**Keywords:** P53, pancreatic cancer, cancer metabolism, gastric cancer, ketogenic diet, ROS, pentose phosphate pathway

## Abstract

Tumor metabolism has emerged as a critical target in cancer therapy, revolutionizing our understanding of how cancer cells grow, survive, and respond to treatment. Historically, cancer research focused on genetic mutations driving tumorigenesis, but in recent decades, metabolic reprogramming has been recognized as a hallmark of cancer. The TP53 inducible glycolysis and apoptosis regulator, or TIGAR, affects a wide range of cellular and molecular processes and plays a key role in cancer cell metabolism by regulating the balance between glycolysis and antioxidant defense mechanisms. Cancer cells often exhibit a shift towards aerobic glycolysis (the Warburg effect), which allows rapid energy production and gives rise to biosynthetic intermediates for proliferation. By inhibiting glycolysis, TIGAR can reduce the proliferation rate of cancer cells, particularly in early-stage tumors or specific tissue types. This metabolic shift may limit the resources available for rapid cell division, thereby exerting a tumor-suppressive effect. However, this metabolic shift also leads to increased levels of reactive oxygen species (ROS), which can damage the cell if not properly managed. TIGAR helps protect cancer cells from excessive ROS by promoting the pentose phosphate pathway (PPP), which generates NADPH—a key molecule involved in antioxidant defense. Through its actions, TIGAR decreases the glycolytic flux while increasing the diversion of glucose-6-phosphate into the PPP. This reduces ROS levels and supports biosynthesis and cell survival by maintaining the balance of nucleotides and lipids. The role of TIGAR has been emerging as a prognostic and potential therapeutic target in different types of cancers. This review highlights the role of TIGAR in different types of cancer, evaluating its potential role as a diagnostic marker and a therapeutic target.

## 1. Introduction

The TIGAR, also known as TP53 inducible glycolysis and apoptosis regulator, plays a critical role in a variety of diseases by influencing a wide range of cellular and molecular processes. This review seeks to outline the conflicting roles of TIGAR, highlighting its function as both a tumor suppressor and promoter, influenced by many factors such as P53, ROS levels, tumor stage, hypoxia, and oncogenic pathways. This review will also clarify TIGAR’s role in directing the cell to undergo PPP instead of glycolysis, resulting in reduced autophagy. First, we will outline the key strategies targeting tumor cell metabolism as a potential adjuvant anti-cancer therapy, hinting at P53 and its downstream effectors like TIGAR. Then, we will focus on its involvement in the metabolic reprogramming and spread of cancer cells, delving into the presence and impact of TIGAR in different types of cancers and emphasizing pancreatic and colorectal cancer. Furthermore, we will explore the potential role of TIGAR as a biomarker for cancer diagnosis and its possible applications as a therapeutic agent. Highlighting the significance of TIGAR in the field of cancer research and treatment due to its role in metabolic reprogramming, oxidative stress regulation, tumor progression, and its dual function in both promoting and inhibiting cancer makes it a complex but promising therapeutic target. Linking key metabolic pathways like glycolysis inhibitors, oxidative phosphorylation inhibitors, glutamine metabolism, and lipid metabolism to TIGAR gives us new insight and links and helps us understand the escalated outcome and progress. Moreover, precision medicine and different diets and amino acid restriction are new phenomena that are elaborated in this review.

This review article aims to contribute to the advancement of our knowledge and potential treatment options for different types of cancer.

## 2. The Emergence of Tumor Cell Metabolism as an Adjuvant Target in Cancer Therapy

Our knowledge of how cancer cells proliferate, endure, and react to therapy has been completely transformed by the discovery that tumor metabolism is a crucial target in cancer therapy. Although genetic changes causing carcinogenesis have been the main focus of cancer research historically, metabolic reprogramming has been identified as a characteristic of cancer in recent decades. Tumor cells exhibit distinct metabolic behaviors, often adapting to sustain rapid proliferation, resist cell death, and thrive in hostile microenvironments, such as hypoxia or nutrient-deprived conditions. These insights have opened new avenues for adjuvant therapies that complement traditional cancer treatments.

### 2.1. The Warburg Effect: Foundational Insights

Otto Warburg first reported the Warburg effect in the 1920s, and it was one of the first findings of abnormal metabolism in cancer cells. Warburg found that even in the presence of enough oxygen, cancer cells preferentially engage in aerobic glycolysis, consuming a lot of glucose and generating lactate. This is in contrast to normal cells, which largely use oxidative phosphorylation for energy synthesis under normoxic conditions. Glycolysis produces the metabolic intermediates required for the production of nucleotides, lipids, and amino acids, meeting the anabolic needs of rapidly proliferating tumor cells, but being less effective than oxidative phosphorylation in terms of ATP generation [1].

Improvements in the field of cancer biology have led to a better understanding of the Warburg effect’s molecular regulators. Tumor suppressor genes like *TP53* and important oncogenes like *RAS* and *MYC* control the metabolic shift toward glycolysis. Furthermore, this glycolytic phenotype is further promoted by hypoxia-inducible factor 1-alpha (HIF-1α) activation in low-oxygen conditions, which strengthens the ability of cancer cells to survive in hypoxic tumor cores [2].

### 2.2. Targeting Key Metabolic Pathways

Over the last few years, many studies investigated exploiting tumor cell metabolic dependencies by targeting critical pathways as an anti-cancer strategy. Several promising metabolic targets have emerged:

#### 2.2.1. Glycolysis Inhibitors

Drugs like 2-deoxyglucose (2-DG) mimic glucose and inhibit glycolysis, starving cancer cells of energy and biosynthetic precursors. However, these agents have shown limited efficacy in clinical settings due to the high metabolic plasticity of tumors, which can switch to oxidative phosphorylation or other pathways when glycolysis is inhibited [3]. Similarly, TIGAR acts as a negative regulator of glycolysis. It decreases intracellular fructose-2,6-bisphosphate (p53 dependent), leading to activation of the pentose phosphate pathway (PPP) and NADPH production, hence its anti-cancer activity [4].

#### 2.2.2. Oxidative Phosphorylation Inhibitors

Targeting mitochondrial metabolism, particularly oxidative phosphorylation, is another approach, especially for cancers that rely on this pathway for energy. Metformin, an antidiabetic drug, has been shown to inhibit complex I of the electron transport chain, limiting ATP production in cancer cells [5]. Interestingly, TIGAR can enhance the expression of the oxidative phosphorylation (OXPHOS) markers in neuronal cells, e.g., peroxisome proliferator-activated receptor gamma coactivator 1 (PGC-1α) and nuclear respiratory factor (NRF1), during neural stem cell differentiation. In this context, TIGAR decreased lactate production and accelerated oxygen consumption and ATP generation to maintain a high rate of OXPHOS in the differentiated cells [6]. There is also evidence that TIGAR influences metabolic processes in cancer cells, including the regulation of oxidative phosphorylation (OXPHOS) markers. In gastric cancer, TIGAR has been shown to inhibit glycolysis and promote antioxidant activities, leading to increased NADPH production, reduced reactive oxygen species (ROS) levels, and enhanced cancer cell survival. This metabolic shift supports mitochondrial function and may indirectly influence OXPHOS pathways [7,8]. Furthermore, PGC-1α expression is enhanced by TIGAR. PGC-1α plays a critical role in cancer metabolism by regulating mitochondrial biogenesis and oxidative metabolism. Its expression is associated with modifications in cancer cell metabolism, impacting tumor growth and patient survival [9]. While TIGAR’s role in modulating glycolysis and maintaining redox balance is established, its specific impact on the expression of PGC-1α and NRF1 in cancer cells requires further investigation.

#### 2.2.3. Glutamine Metabolism

The phenomenon of “glutamine addiction” in tumor cells is an area of active research, as cancer cells frequently rely on glutamine as a key metabolic substrate for growth and survival. Glutaminase (GLS) inhibitors, such as CB-839, have shown significant promise in preclinical studies by blocking the conversion of glutamine to glutamate, thereby depriving tumor cells of essential substrates for biosynthesis and energy production. These inhibitors are currently being tested in clinical trials and have demonstrated potential for enhancing the efficacy of other cancer therapies by inducing metabolic stress within tumor cells. Moreover, targeting glutamine transporters and other related metabolic pathways also represents a promising avenue to weaken cancer cell survival mechanisms [10,11]. DNA damage-induced transcript 3 (DDIT3) exerts an adaptive survival mechanism of cancer cells to encounter glutamine starvation by balancing glycolysis and oxidative phosphorylation. DDIT3 is induced during glutamine deprivation to promote glycolysis and adenosine triphosphate production via suppression of TIGAR, the negative glycolytic regulator [12].

#### 2.2.4. Lipid Metabolism

Lipid biosynthesis and β-oxidation are frequently upregulated in various cancers. As a result, targeting critical enzymes involved in these pathways—such as fatty acid synthase (FASN) in fatty acid synthesis and carnitine palmitoyltransferase 1A (CPT1A) in β-oxidation—is being actively investigated as a potential therapeutic approach. Inhibiting these enzymes could disrupt cancer cell metabolism and inhibit tumor growth [13,14]. Intriguingly, TIGAR is involved in macrophage foam cell formation and atherosclerosis development [15]. This may be reflected in the macrophage function and role in the tumor microenvironment.

### 2.3. Nutrient Deprivation and Dietary Interventions

**Figure 1:**
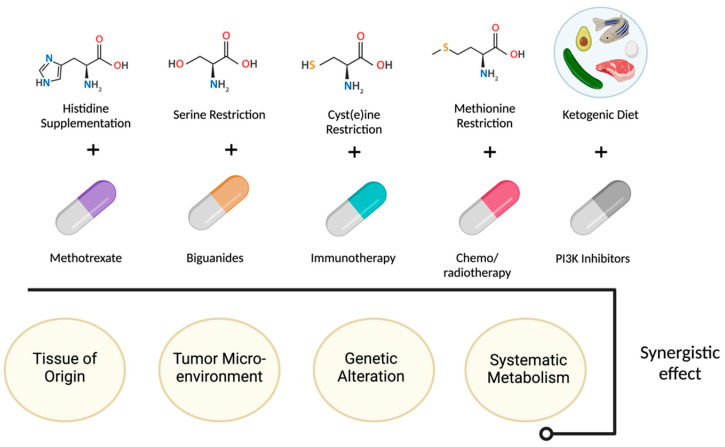
Nutrient deprivation and dietary interventions effect.

Another approach to targeting tumor metabolism is through nutrient deprivation or dietary interventions. Tumor cells often have a higher demand for specific nutrients, and restricting their availability can impair tumor growth, e.g., limiting certain amino acids. Also, the ketogenic diet, which severely restricts carbohydrate intake, has been investigated as an adjunct therapy. Nutrient deprivation along with certain drugs are summarized in Figure 1. By forcing the body to rely on fat-derived ketones instead of glucose for energy [16].

### 2.4. Immunometabolism: The Tumor Microenvironment

Targeting the metabolic interplay between tumor cells and the immune system is a promising strategy for enhancing cancer immunotherapy. The tumor microenvironment (TME), composed of immune cells, fibroblasts, and the extracellular matrix, plays a crucial role in tumor progression. Cancer cells can manipulate the metabolic landscape of the TME, creating an immunosuppressive environment that dampens anti-tumor immune responses. Metabolic competition between tumor cells and immune cells, particularly T-cells, can deprive immune cells of essential nutrients like glucose and amino acids, impairing their function. Modulating the metabolism of T-cells to make them more resilient in the nutrient-poor, immunosuppressive TME can improve their anti-tumor activity [17,18]. Reactive oxygen species (ROS) can both promote and suppress malignant progression, depending on the tumor type and stage of cancer development. Recently, Cheung et al. showed that ROS levels in cancer cells impact their interaction with surrounding normal stromal cells, with higher ROS in cancers with TIGAR knock down inducing a more tumor-supportive behavior of surrounding fibroblasts and macrophages. TIGAR deficiency, which leads to more ROS, increases metastasis in a mouse model of pancreatic cancer, and overexpression of TIGAR (resulting in less ROS) decreases tumor invasiveness [19].

## 3. TIGAR: The Gene and the Protein

The *TIGAR* gene is pleiotropic (can give rise to many different phenotypes, which usually play a crucial role in many diseases). The *TIGAR* gene (around 38,835 bp), which encodes the TIGAR protein (813 bp), is highly conserved in humans and animals, and it is located on chromosome 12p13.32; it is also known as C12orf5, and it consists of six coding exons. The end product of the translated mRNA represents a unique intracellular protein with a 30 kDa molecular weight and a chain of 270 amino acids. There are 878 identified SNP variants in the *TIGAR* gene, some of which have been detected in patients and are associated with phenotypic changes that may influence TIGAR’s function in cellular metabolism and response to stress [20]. TIGAR SNPs identified on the COMIC website were also added to the Supplementary Material (Figure S1) [21]. The TIGAR protein structure has common secondary structural elements such as alpha helices and beta sheets and predicted intrinsic flexibility. Under hypoxic conditions, TIGAR has a known interaction with hexokinase 2 (HK2) on the outer mitochondrial membrane. This binding boosts HK2 activity, facilitating glucose phosphorylation and helping to sustain mitochondrial membrane potential, which in turn lowers ROS levels and inhibits apoptosis [22]. The TIGAR protein belongs to the phosphoglycerate mutase family, which under the catalytic reaction will be phosphorylated; in addition, it plays a role in substrate hydrolysis [23].

Although TIGAR is normally expressed in almost all tissues like muscle, brain, and heart, on the other hand, it is also highly expressed in Alzheimer’s [24] and some types of tumors such as colon, breast, and pancreatic cancer. However, detailed quantitative data on relative expression levels across different tissues are not provided in the current literature. Moreover, TIGAR protein levels are extremely high in the embryonic state, and they moderately decrease with aging. The expression of TIGAR in normal tissue and different types of cancer is demonstrated in Figure S2 [25]. The *TP53* tumor suppressor target gene has a major role in glucose metabolism and autophagy. The role of TP53 in cancer is summarized in Figure 1. An overview of its regulation is also demonstrated. The expression of TIGAR protects cells from ROS by decreasing DNA damage-induced apoptosis. TIGAR hinders autophagy by blocking glycolysis and directing the pathway to the pentose phosphate pathway, which upgrades NADPH production and prevents oxidative stress in several diseases [26,27,28].

### 3.1. The Role of TIGAR in Cancer

TIGAR is a fructose-2,6-bisphosphatase. In vitro, it also has fructose-1,2-bisphosphatase, in addition to 2,3-bisphosphoglycerate phosphatase activity. TIGAR can decrease fructose-2,6-bisphosphate (F-2,6-P2), an allosteric activator of phosphofructokinase-1 (PFK-1) in the glycolytic pathway, thus decreasing PFK-1 activity and potentially shifting the glucose metabolism to the pentose phosphate pathway (PPP) with the production of the antioxidant protective molecule NADPH [28].

While TIGAR shares structural homology with fructose-2,6-bisphosphatases (FBPases) of the PFKFB family, it exhibits relatively low catalytic efficiency compared to classical FBPases, but this ensures a controlled shift towards antioxidant pathways, protecting cells from oxidative stress rather than driving fast energy production [28,29].

Although the detailed effects of TIGAR expression on metabolism remain to be determined, it is clear that TIGAR functions in many cell systems to mediate antioxidant defense mechanisms through an increase in NADPH and GSH [30].

TIGAR also suppresses autophagy. Consequently, TIGAR might boost cancer cell survival in response to chemotherapy by blocking both apoptosis and autophagy. TIGAR has been found to play an important role in the development of invasive primary cancers by inhibiting oxidative stress. The expression of the *TIGAR* gene is dynamically regulated during the advancement of pancreatic ductal adenocarcinoma; decreased levels of ROS promote tumor initiation in the premalignant condition, and increased levels of ROS can enable metastatic progression [31].

**Figure 2:**
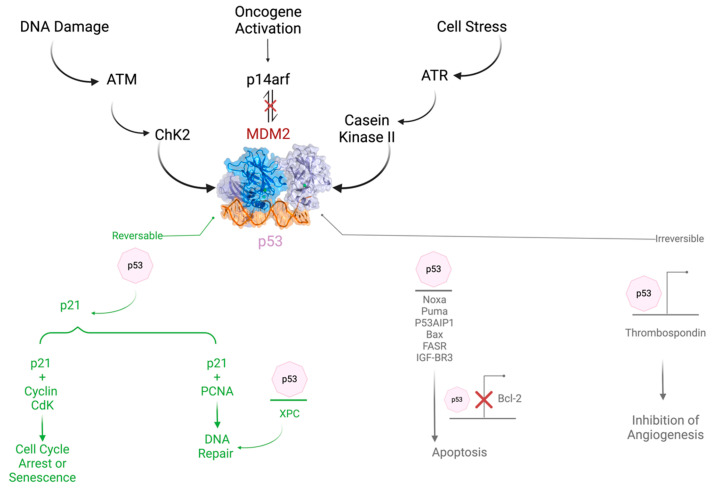
The role and regulation of TP53 in cancer.

TIGAR limits ROS and supports primary tumor development. The role of TIGAR is summarized in Figure 2.

**Figure 3:**
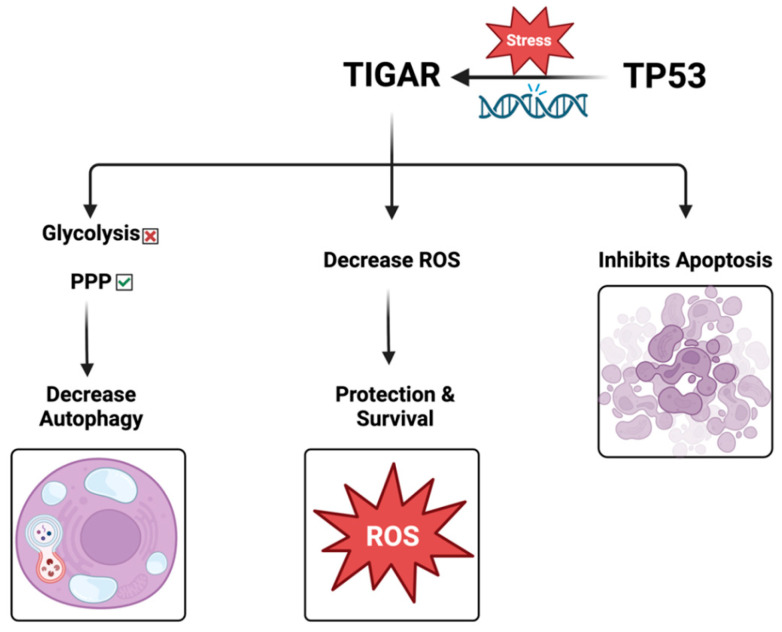
The role of TIGAR protein.

TIGAR loss promotes metastasis and enhances invasion. Accordingly, the role of ROS varies during tumor progression. TIGAR increases in the initial stages of cancer, consistent with its role in hindering ROS and encouraging the survival of the preinvasive cells. Nonetheless, the progression to invasive tumors is accompanied by a noticeable decrease in TIGAR expression, and the ROS levels are markedly increased, promoting the signaling of mitogen-activated protein kinase (MAPK) by reducing dual-specific phosphatase-6. These effects drive the dynamic transformation of cancer from a proliferative type to an invasive type, thereby enhancing the ability of migration, invasion, and metastasis [7]. Therefore, TIGAR-mediated changes in ROS might be a vital mechanism for regulating the occurrence and progression of many cancers (Figure 3).

Noteworthy, TIGAR is overexpressed in many cancers. Its expression is associated with a dismal prognosis, whereas knocking out TIGAR in experimental animal models leads to better survival. Chu et al., 2020, showed that TIGAR is a major player in esophageal squamous cell carcinoma (ESCC) progression and chemoresistance [30,32]. TIGAR reprograms glucose metabolism from glycolysis to the glutamine pathway through AMP-activated kinase. Its overexpression is correlated with poor prognosis. Knocking out *Tigar* in mice has reduced ESCC growth and proliferation. Treatment of *TIGAR*-overexpressing ESCC cell xenografts and patient-derived tumor xenografts in mice with a combination of glutaminase inhibitor and chemotherapeutic agents achieves significantly more efficacy than chemotherapy alone [30]. Interestingly, TP53 is known to be involved in redox balance; any dysregulation of its activity contributes to oxidative stress. Table 1 summarizes the different factors affecting TIGAR.

#### 3.1.1. TIGAR in Cancer Development and Progression: A Potential Diagnostic and Prognostic Biomarker

TIGAR expression deregulation was shown to be relevant in oncogenesis and cancer progression [26,29,36,37,38,39].

For instance, in pancreatic cancer, TIGAR supports premalignant tumor initiation [31]. Moreover, in CRC, TIGAR protein levels were significantly upregulated in later stages [40].

Understanding TIGAR expression in different cancer types and its relationship with patients’ survival rate can be an attractive tool for cancer therapeutics and useful for clinical diagnosis and prognosis.

However, due to the very limited number of studies done of TIGAR, it is hard to have data regarding the sensitivity and specificity. The application of TIGAR as a biomarker faces several challenges. Firstly, the absence of validated and standardized assays for measuring TIGAR levels impedes its integration into routine clinical practice. Secondly, TIGAR’s expression varies across different cancer types and stages, affecting its reliability and consistency as a universal biomarker. Lastly, there is insufficient clinical validation to support TIGAR’s sensitivity and specificity in cancer detection or prognosis.

The p53 tumor-suppressor protein employs multiple mechanisms to hinder the proliferation of damaged cells and cancer development through inducing cell-cycle arrest and apoptosis. TIGAR stands out as a well-recognized protein regulated by p53, mediating its effect on metabolism [41]. TIGAR’s expression reduces fructose-2,6-bisphosphate concentrations within cells, therefore leading to the inhibition of glycolysis and shifting the metabolic flux to the PPP, producing NADPH and ribose-5-phosphate, which are crucially required by rapidly dividing cells to replicate their DNA. The overall decline in levels of ROS within cells due to TIGAR expression can affect p53’s ability to guard against ROS-associated cell damage, tumor development, and apoptosis [42]. Studies have shown that upon the knockdown of endogenous TIGAR expression, p53-induced cell death is enhanced, indicating that TIGAR’s expression might fine-tune the response to p53-induced apoptosis, offering protection when exposed to mild or transient stress signals [41]. TIGAR, an enzyme involved in glycolysis and a participant in antioxidant responses, often associates with an aggressive tumor phenotype and plays a crucial role in establishing resistance to chemotherapies. It is overexpressed in many types of cancer and can be used as an indicator of poor prognosis, advanced tumor growth, and determinant of therapy responsiveness [43].

##### Pancreatic Cancer

Using a pancreatic ductal adenocarcinoma (PDAC) model, researchers found that ROS regulation by TIGAR supports premalignant tumor initiation while restricting metastasis. Increased ROS in PDAC cells drives a phenotypic switch that increases migration, invasion, and metastatic capacity. This switch can be reversed by treatment with antioxidants, which increases activation of the MAPK signaling pathway. In both mice and humans, TIGAR expression is strongly affected by PDAC development, with higher levels of the protein present in premalignant lesions and lower levels in metastasizing tumors. It is clear that temporal and dynamic control of ROS is critical for full malignant progression, and this information helps to explain conflicting reports on the pro- and anti-tumor effects of antioxidant treatment. To better understand the role of TIGAR in the development of pancreatic cancer, researchers used well-established mouse models with different mutation profiles (KC), (KPC), or (KFC) [44,45,46]. In the KFC model, one of the copies of the *p53* gene is deleted by CRE-Lox recombination, leading to the loss of the remaining copy of the gene during tumor development (Table 2) [31].

Each model was crossed with a Tigarfl/fl strain to generate pancreatic tumors that retained *TIGAR* expression (CTR) or deleted (KO) for TIGAR. These tumor models were then used to study the effects of TIGAR on pancreatic cancer. Initial analysis of preneoplastic PanIN in the KC model demonstrated that loss of TIGAR delayed the representation of each stage of PanIN progression (PanIN1, 2, and 3), accompanied by lower proliferation in the TIGAR null lesions, measured by Ki67 staining. Using the KFC model, PanIN lesions were intensely detected, resulting in the loss of TIGAR, which retarded the appearance of PanIN and lowered the proliferation of these preneoplastic lesions.

The results of this study support previous research that suggests a loss of the *TIGAR* gene can delay the appearance of intestinal adenomas in response to a loss of the *APC* gene, as well as work that suggests a decrease in the development of PanIN cells following a loss of the antioxidant factor NRF2 in a prostate cancer model. Anti-malondialdehyde (MDA) staining was used to measure oxidative stress in the PanIN cells from TIGAR KO mice and found that there was an increase in ROS in the TIGAR KO PanINs (in the KC and KFC models) as well as TIGAR KO PDAC cells (in the KFC model). There is evidence that TIGAR plays an antioxidant role and that this may be related to increased levels of ROS in the cells. Treatment with an antioxidant, N-acetyl-L-cysteine, can reduce these levels. The TIGAR KO cells showed increased death following exposure to the chemotherapeutic agent adriamycin (doxorubicin), which was limited by treatment with the antioxidant NAC. These results suggest that the TIGAR KO cells are more sensitive to the effects of ROS than normal cells. Importantly, introducing recombinant TIGAR into TIGAR null cells lowered ROS levels, which also rescued the sensitivity to Adriamycin.

TIGAR has been shown to support flux through the oxidative PPP, which generates NADPH for antioxidant defense. This NADPH can then be used to support the cellular antioxidant defense system. Both oxidative and non-oxidative ribose-5-phosphate (R5P) production are seen in PDACs, and previous studies have shown that mutant KRAS-expressing tumors generate more ribose-5-phosphate through the non-oxidative pathway. There were no differences in the levels of R5P proteins between *TIGAR* wild-type and null cells, suggesting that in TIGAR null cells, there is a defect in oxidative PPP, which is likely to be compensated for by an increase in non-oxidative PPP flux. Therefore, the results demonstrate that TIGAR limits oxidative stress, a function that corresponds with the ability of TIGAR to support the initial stages of PDAC development [37].

Interestingly, during cancer progression, dynamic changes in TIGAR expression are observed. Immunohistochemical analysis of TIGAR at various stages of PDAC tumorigenesis shows an increase in TIGAR expression during the early stages of tumor progression in both the KFC mouse model and human PDAC samples. TIGAR may play a role in limiting the levels of ROS in cells and promoting their survival. This may be important in preventing preinvasive cells from becoming invasive. However, in both mouse and human cancers, progression to invasive primary tumors was accompanied by a clear decrease in TIGAR expression. The levels of ROS in PDAC lesions were correlated with the levels of TIGAR, with higher ROS levels in the later stage. The higher ROS levels in late-stage invasive tumors suggest that these tumors may have lower levels of the tumor-inhibiting gene [31].

##### Gastric Cancer

Gastric cancer (GC) remains the fifth most common malignant tumor and the third leading cause of cancer-related death worldwide [47]. Despite the remarkable progress in GC early detection and treatment, most patients have already metastasized or progressed to high malignancy when diagnosed [48]. Thus, searching for early diagnostic and prognostic biomarkers is highly required.

Recently, Zhijuan et al. investigated a prognostic risk model to identify reliable biomarkers that might contribute to a better GC prognosis. Their study was based on using data from The Cancer Proteome Atlas (TCPA) and The Cancer Genome Atlas (TCGA) [49]. The study screened 218 proteins, where 15 different proteins, including TIGAR, were identified as significantly related to the overall survival of GC patients. Seven proteins were listed as protective proteins, including NDRG1_pT346, SYK, P90RSK, TIGAR, and XBP1 [49]. These findings indicate that TIGAR protein can be used as a prediction marker for gastric cancer, where its expression implies gastric cancer development and progression.

These findings are consistent with another study, conducted by Guo-Liang et al., where they identified TIGAR along with two other proteins, collagen VI and CD20, as independent prognostic factors. This study was based on the Cancer Proteome Atlas (TCPA) and used machine learning. Two categories of patients were defined: a high-risk group and a low-risk group. TIGAR was identified as a low-risk marker. Furthermore, its expression was positively correlated with overall survival [50].

##### Colorectal Cancer

Colorectal cancer results from both genetic and epigenetic modifications in epithelial cells, leading to their transformation into cancerous cells and further their metastasis to distant organs [51]. Despite the advances in early diagnosis and treatment, CRC remains the leading cause of cancer-related death worldwide [40,51]. Therefore, there is an urgent need to identify markers allowing the detection of CRC at early stages. In 2015, TIGAR expression and its correlation with the various cancer stages were investigated. TIGAR expression was determined via tissue microarray, immunohistochemistry, and protein expression from several matched colorectal tumor tissues and adjacent normal tissues [52]. TIGAR mRNA level was significantly upregulated in stage II compared to normal tissue. Immunochemistry results showed an increased TIGAR expression in CRC. The protein analysis was consistent with the previous findings indicating an increased protein expression in both stages II and III. Altogether, these data support the use of TIGAR expression as a biomarker for CRC detection and a potential therapeutic target for CRC treatments [52].

In another study, the diagnostic utility of TIGAR in both CRC and benign bowel diseases was investigated. For this purpose, 180 tissue samples were collected and divided into three groups (CRC, non-neoplastic colon tissue, and inflammatory bowel disease). Both mRNA and protein levels of TIGAR were determined to evaluate its expression level in the three mentioned groups. An upregulation of TIGAR in CRC tissues and benign colonic lesions was detected compared to non-tumor tissues. A significant correlation was detected between TIGAR expression at a protein level, TNM staging, and metastasis. TIGAR was overexpressed in CRC tissues compared to control tissues and benign lesions at both mRNA and protein levels. *TIGAR* gene upregulation was associated with TNM staging and with the presence of lymph node metastasis. Given all that, they address the use of TIGAR as a potential biomarker for CRC diagnosis and staging [53].

##### Nasopharyngeal Carcinoma

Nasopharyngeal carcinoma (NPC) is one of the head and neck malignancies, with high incidence in East and South Asia (up to 71%). Despite the improved patients’ survival rate due to the current treatments, intensity-modulated radiation therapy and adjuvant chemotherapy, clinical failure is always a major concern due to local recurrence and distant metastasis [40,51]. Several biomarkers have been identified to predict a favorable prognosis in NPC [54,55]; identifying more potent markers will add value, especially to help in prognosis prediction. Min Wei et al. investigated the correlation between autophagy and the expression levels of TIGAR and their association with clinical outcomes in NPC patients.

A previous report investigated the relationship between TIGAR and microtubule-associated protein 1 light chain 3 (*LC3B*). Results show that the positive TIGAR expression is associated with poor prognosis, and examining the different patterns of TIGAR and LC3B in NPC tumors revealed that TIGAR+ and LC3B− expressions alone and in combination were highly associated with poor prognosis. Overall, these data hint at the potential use of TIGAR and LC3B as indicators in NPC patients [39].

##### Renal Cell Carcinoma

Renal cell carcinoma (RCC) is the most common type of kidney cancer in adults and accounts for about 90–95% of cancerous cases arising from the kidney [56,57]. Most RCC cases are asymptomatic with non-specific symptoms, including weight loss and fever. Only 15% of the RCC cases present the classic triad of hematuria, flank pain, and flank mass at the time of diagnosis, and unfortunately, it is a sign of an advanced disease [58]. Nearly 30% of RCC patients were reported to have metastatic spread at the time of diagnosis [59].

Clear RCC is the most common histologic type in RCC, accountable for nearly 70% to 80% of RCC cases [60]. Patients with clear RCC have a worse prognosis compared to the other histological types, with only a 5-year survival [60,61,62].

The maximal standardized uptake value (SUVmax) is a robust metric for the in vivo assessment of ^18^F-fluorodeoxyglucose (FDG) uptake and glucose metabolic activity of tumors [63]. Studies have shown an association between SUVmax values and RCC prognosis [64,65], indicating that SUVmax may provide a quantitative measurement of glucose metabolism of tumor lesions and predict disease prognosis in patients with RCC. In a recent retrospective study conducted by Xiaoyan et al., researchers tried to investigate the potential role of TIGAR expression and SUVmax in patients with clear cell RCC as a predictive factor for disease survival in clear RCC patients [29]. In addition, they tried to establish a correlation between TIGAR expression and ^18^F-FDG PET/CT imaging parameters in patients with clear cell RCC, knowing that ^18^F-fluorodeoxyglucose positron emission tomography/computerized tomography [^18^F-FDG PET/CT] is one of the most important functional imaging techniques used for tumor diagnosis, metastasis detection, and staging in various cancers [66,67]. Interestingly, TIGAR expression and high SUVmax were associated with poor prognosis, concluding that patients with positive TIGAR expression and high SUVmax have worse disease survival [29].

##### Esophageal Squamous-Cell Carcinoma

Esophageal squamous cell carcinoma (ESCC) is primarily treated with surgery (esophagectomy) [68,69], and for locally advanced ESCC, chemotherapy and radiotherapy are used, but the survival rates are low [70,71,72]. A better understanding of the molecular mechanism of ESCC is needed to overcome the lack of effective ESCC treatment and prevention. TIGAR overexpression has been associated with different human cancer types [23], and TIGAR relevance was never assessed in ESCC. For this purpose, Jiahui Chu et al. investigated the role of TIGAR in ESCC progression and resistance to chemotherapy in 225 patients. All patients underwent esophagectomy without any chemotherapy or radiotherapy. Adjacent normal tissues were also collected from each of the patients for analysis. They have found that TIGAR is overexpressed in most human ESCC samples. Additionally, they have found that TIGAR expression levels are significantly correlated with advanced tumor stages, lymph node metastasis, and poor patient survival. Overall, this study highlights the possibility of using TIGAR expression levels as a biomarker in ESCC [32].

##### Acute Myeloid Leukemia

Acute myeloid leukemia (AML) is a heterogeneous group of hematopoietic disorders with different clinical outcomes [73]. The cytogenetically normal AML (CN-AML) constitutes the largest subset, representing 45–60% of all AML cases [23,41]. The role of TIGAR in CN-AML was investigated by Sixuan Qian et al., who showed that *TIGAR* knockdown led to the suppression of human leukemic cell proliferation [74].

##### Glioblastoma

On a worldwide scale, glioblastoma is the most common type of malignant brain cancer in adults [75]. Despite the presence of advanced techniques developed to treat glioblastoma, such as advanced surgical techniques, radiotherapy, and chemotherapy drugs, glioblastoma is still associated with a poor prognosis with only a 5-year survival rate of 5–13% [76]. TIGAR expression was measured in glioma samples and cell lines by Western blot and immunochemistry, and its role was assessed in migration, invasion, and EMT (epithelial to mesenchymal transition). An upregulation of TIGAR was observed in glioblastoma associated with poor survival. In addition, TIGAR knockdown had a drastic effect on migration, invasion, and EMT [35].

##### Non-Small Cell Lung Cancer

Non-small cell lung cancer (NSCLC) includes a variety of different lung cancers such as adenocarcinoma, squamous cell carcinoma, and large cell carcinoma, with adenocarcinoma being the most common type of lung cancer [35]. In order to understand the role of TIGAR and its potential use as a biomarker in NSCLC, an immunohistochemical analysis of 72 NSCLC patients was conducted, and it showed that both TIGAR and Met expression correlated positively with late stages of lung cancer. Met is encoded by the MET proto-oncogene, and its expression was found to be correlated with enhanced proliferation, motility, invasiveness, and angiogenesis in NSCLC [77]. Furthermore, TIGAR was found responsible for the NSCLC cells EMT phenotype through the activation of mesenchymal markers and downregulating epithelial markers. TIGAR knockdown resulted in a reduced expression of both metalloproteinases MMP2 and MMP9, known for their role in tumor microenvironment formation and cancer progression and metastasis. These findings highlight the importance of the TIGAR/Met pathway in NSCLC and suggest this pathway for novel targeted therapy [77].

##### HPV18 Cervical Adenocarcinoma

Zhang et al. have linked TIGAR upregulation with an aggressive cervical cancer phenotype in patients using fluorine-18-fluorodeoxyglucose PET/computed tomography scans [78]. They checked the expression levels of TIGAR protein by screening HPV16+ cervical cancer clinical isolates by confocal microscopy. As expected, TIGAR was overexpressed in these isolates. Inhibition of TIGAR led to more sensitive HPV18-transformed HeLa cells towards conventional chemotherapeutic agents doxorubicin, cisplatin, etoposide, and cyclophosphamide. These findings relate HPV16+ cervical cancer cell resistance to conventional chemotherapy to TIGAR overexpression. This suggests TIGAR’s inhibition as a strategy to sensitize hrHPV+ tumor cells to chemotherapy agents [78].

#### 3.1.2. TIGAR as a Potential Therapeutic Target in Treating Different Types of Cancers

TIGAR modulation has been suggested as an effective tool in the therapeutic strategy for a myriad of cancers. Interfering with the protein can successfully enhance the effect of other medications and combat the aggressiveness of the cancer cell.

##### Non-Small Cell Lung Cancer

TIGAR is located in the cytoplasm and can enter organelles, including the mitochondria, forming a complex with HK2. HK2 plays an important role in glycolysis and apoptosis; it is known to catalyze the phosphorylation of glucose, which is the first step of glycolysis. In non-small lung cancer (NSLC) cells, HK2 inhibits apoptosis by restraining the changes that occur to the mitochondrial membrane permeability during the process. In a previous study, it was proven that following the combined exogenous p53 and cisplatin treatment, the expression of TIGAR was suppressed; therefore, the glucose flux shifted from the PPP to glycolysis, reducing ATP production and altering metabolic patterns. The localization of the TIGAR-HK2 complex in the mitochondria was also reduced, increasing the mitochondrial pathway apoptosis, therefore increasing the sensitivity of NSLC cells A459 to cisplatin [79].

##### Esophageal Squamous Cell Carcinoma

TIGAR is also known for playing an important role in the progression of ESCC and resistance to chemotherapy. TIGAR orchestrates a shift in glucose metabolism, diverting it from glycolysis to the glutamine pathway by reducing F-2,6-BP; TIGAR decreases glycolytic flux, shifting the cell’s reliance from glucose to glutamine using AMP-activated kinase. It was shown that by knocking down TIGAR expression in ESCC cell lines, significant suppression of the malignant phenotypes of the cells was evident. TIGAR knockout in mice models was also tested, showing a significant reduction in ESCC tumor size and growth rate; furthermore, the migration and invasion were suppressed in the ESCC cell line. Since the overexpression of TIGAR inhibits glycolysis, cancer cells tend to use a compensatory energy-providing pathway to increase ATP production and sustain the survival and progression of ESCC via activating AMPK. Hence, inhibiting the glutamine pathway increases the sensitivity to cytotoxic chemotherapeutic agents. Administering a combination of a glutaminase inhibitor and 5FU/DDP to mice with TIGAR-overexpressing ESCC cell grafts and patient-derived tumor grafts led to significant suppression of tumor growth compared to using chemotherapy alone. The effect of this combination was also tested on normal tissues, showing minimal systemic toxicity [32].

##### HPV18-Transformed HeLa Cervical Adenocarcinoma

Anti-cancer drugs are known to cause damage to other tissues, particularly when administered in high doses; hence, constraining their potential applications [80]. Doxorubicin is one of the most effective anti-cancer drugs known to cause DNA damage and ROS generation. Its clinical use is limited due to the adverse effects that are developed in cancer patients, mainly cardiotoxicity [81]. HeLa cells are known to form significant resistance to apoptosis induced by doxorubicin. Similar results were found when TIGAR was knocked down in HPV18-transformed HeLa cervical adenocarcinoma cells treated with different doxorubicin concentrations inducing a significant increase in cytotoxicity and dose-dependent apoptosis compared to scrambled RNA negative control and fibroblast cell line. HeLa cells were hypersensitized to low-dose chemotherapeutic drugs, enhancing oxidative stress, ROS accumulation, and DNA damage. These outcomes could lead to improved clinical results by reducing the toxic side effects caused by doxorubicin, therefore rendering it more acceptable by patients [43].

##### Myeloid Leukemia

TIGAR is overexpressed in many types of cancers, including leukemia, lung cancer [82], colon cancer [26], and liver cancer [83]. TIGAR protects cancer cells from programmed cell death, supporting their progression and growth through inhibiting glycolysis; therefore, shifting metabolism to the pentose phosphate pathway and producing NADPH and glutathione as antioxidants and ribose-5-phosphate for nucleotide synthesis. The function of TIGAR in glycolysis and survival of acute myeloid leukemia cells was studied in vitro and in vivo. It was found that the high expression of TIGAR in patients with acute myeloid leukemia can be a predictor of poor survival and a high incidence of relapse. In vivo study confirmed that the knockdown of TIGAR leads to the inhibition of the proliferation of cancer cells and increases the sensitivity of these cells to the anti-glycolytic agent 2-deoxy-D-glycose, therefore increasing the apoptosis of leukemia cells in the xenograft mouse model. These results confirm that TIGAR is important for glycolysis of leukemia cells. More studies should be done to discover specific TIGAR inhibitors; hence, the combination of glycolytic inhibitors with TIGAR inhibitors along with the chemotherapeutic agents that are already being used today can be a potential and effective treatment for myeloid leukemia [74].

##### Glioblastoma

Glioblastoma is known to be associated with poor prognosis, chemotherapy resistance, and rapid growth through migration and invasion of cancer cells to neighboring tissues [84]. It has been shown that TIGAR is overexpressed in glioblastoma patients’ tissues as well as in the glioblastoma cell line U-87MG. TIGAR overexpression in patients was inversely correlated with the survival time of patients with glioblastoma. TIGAR knockdown results in an increase in ROS production and a decrease in cell viability due to oxidative stress and an increase in NADPH. Glioblastoma is known to be resistant to Tomzolomide, an alkylating agent used to cause DNA damage. Upon TIGAR knockdown and TMZ treatment, anti-apoptotic bcl-2 protein was decreased with an increase in bax pro-apoptotic protein, implying the increase in apoptosis in these cells in addition to having a significant effect on migration and invasion of glioblastoma cells. TIGAR can be considered as a potential target for treating glioblastoma [35].

Table 3 summarizes the different types of cancer that are affected by the over-expression of TIGAR protein and the novel potential therapies that can be further studied to be used clinically.

## 4. Conclusions and Future Perspectives

In conclusion, the multifaceted role of the *TIGAR* gene and its associated protein in cellular processes in the context of cancer biology. TIGAR, regulated by the tumor suppressor p53, emerges as a critical player influencing metabolism, redox balance, and cellular responses to stress. Its ability to modulate glycolysis and redirect metabolic flux toward the PPP contributes to the production of essential molecules required for cell proliferation.

TIGAR’s impact on ROS levels has significant implications for cancer development. By reducing ROS, TIGAR can affect p53’s ability to protect against DNA damage, tumor initiation, and apoptosis. Notably, TIGAR’s role in fine-tuning the response to apoptosis suggests its involvement in cellular decisions regarding survival under different stress conditions.

In the context of cancer, TIGAR’s overexpression is associated with aggressive tumor phenotypes and chemoresistance, making it a potential biomarker for poor prognosis and advanced tumor growth. Moreover, its dynamic regulation during different stages of cancer progression, with decreased expression in invasive tumors, underscores its role in the complex landscape of tumor development and metastasis.

Furthermore, TIGAR’s role in metabolic pathways, along with the potential therapeutic strategies such as methionine and cysteine restriction and others, highlights the intricate connections between cellular metabolism and cancer progression. Targeting TIGAR may offer a promising approach to modulating cancer cell behavior, enhancing the effectiveness of existing therapies, and influencing patient outcomes. In addition, it is remarkable that the dietary pattern of cancer patients could play an important role in determining the course and outcome of the disease. The link between the available nutrients exploited by the cancer cell and its aggressiveness has recently become of great impact on the clinical side.

Overall, the comprehensive understanding of TIGAR’s functions in cancer biology, its association with various signaling pathways, and its potential as a biomarker and therapeutic target positions it as a crucial player in the intricate network of molecular events contributing to cancer initiation, progression, and response to treatment. Further research and clinical investigations will likely unveil additional details, ultimately paving the way for innovative and targeted cancer therapies.

## Figures and Tables

**Table 1:** Factors affecting TIGAR.

Factors	Tumor Suppressor	Tumor Promotor
P53 status	Functional p53: TIGAR is upregulated by wild-type p53, leading to reduced reactive oxygen species (ROS) and protection against DNA damage, thereby inhibiting tumor initiation [23].	Mutant/loss of p53: Despite p53 mutations, TIGAR can be overexpressed through alternative pathways, aiding in cancer cell survival by enhancing antioxidant defenses [7].
ROS levels	By lowering ROS levels, TIGAR prevents oxidative stress-induced damage, contributing to tumor suppression [23].	In established tumors, TIGAR-mediated ROS reduction supports cancer cell survival and proliferation [33].
Tumor stage	Early-stage: TIGAR’s antioxidant function helps maintain genomic stability, preventing early tumor development [23].	Advanced stage: Cancer cells exploit TIGAR to adapt to metabolic stress, promoting tumor progression [33].
Hypoxia	Under normal conditions, TIGAR maintains redox balance, supporting normal cellular functions [23].	In hypoxic tumor microenvironments, TIGAR is upregulated, aiding cancer cells in coping with low oxygen levels [34].
Oncogenic pathways	In the absence of oncogenic signals, TIGAR functions align with p53-mediated tumor suppression [23].	Oncogenes like *c-Met* can upregulate TIGAR, enhancing glycolysis and NADPH production, thus supporting rapid tumor growth [35].

**Table 2:** Mouse models used in TP53 and TIGAR studies [44].

Mouse	Gene	Mutation	Generation Method
KC	*LSL-*	KRAS (KrasG12D/+)	A G12D mutation is introduced in exon 2 of the mouse endogenous KRAS allele.
KPC	*LSL-*	KRAS (KrasG12D/+) + p53 (p53R172H/+)	A conditional point-mutant allele of the transformation-related protein 53 gene (p53R172H; structural mutant homologous to human p53 codon 175). The conditional allele is functionally equivalent to a null mutation.
KFC	*P53fl/+*	Mutant KRAS with loss of p53	A mouse model that has one copy of the *p53* gene flanked by loxP sites and one copy of the *p53* gene that is not flanked by loxP sites.

**Table 3:** Potential role of TIGAR as a therapeutic target in different types of cancer.

Type of Cancer	Novel Potential Therapy	Outcome
Non-small cell lung cancer	Exogenous p53+cisplatin combination resulted in	Increased sensitivity of A549 to cisplatin [79].
Esophageal squamous cell carcinoma	TIGAR knockdown led to decreased aggressive disease phenotype in the ESCC cell line, and the combination of glutaminase inhibitor with 5FU/DDP showed significant suppression in tumor growth [32].	Significant suppression of the malignant phenotypes in ESCC cell lines.
HPV18-transformed HeLa cervical adenocarcinoma	The knockdown of TIGAR in HeLa adenocarcinoma cells hypersensitized cancer cells to low doses of doxorubicin, improving the patients’ quality of life [43].	Increased sensitivity of HeLa cells to doxorubicin.
Myeloid leukemia	TIGAR knockdown sensitized leukemia cells to the glycolysis inhibitor 2-DG, therefore increasing apoptosis in-vivo [74].	Increased sensitivity of leukemia cells to anti-glycolytic agent 2-deoxy-D-glycose.
Glioblastoma	TIGAR knockdown led to a decrease in cell viability and the combination with Tomzolomide increased apoptosis and decreased invasion and migration [35,84].	Combination with TMZ treatment increased apoptosis and decreased migration of glioblastoma cells.

## Data Availability

No new data were created in this study.

## Supplementary Materials

The following supporting information can be downloaded at: https://www.mdpi.com/article/10.3390/cells14080598/s1, Figure S1: TIGAR Single nucleotide polymorphisms (SNPs) identified on the COMIC website A. Summary of types of mutations observed in the TIGAR gene. B. Detailed representation of the substitution mutations observed in the TIGAR gene. Figure S2: TIGAR expression in cancer versus normal tissues. nRPX is calculated as log2(intensity), derived from global protein abundance measurement at the protein level. These measurements are obtained through mass spectrometry performed by CPTAC on tissues using isobaric tandem mass tags (TMT). Retrieved through (https://www.proteinatlas.org/ENSG00000078237-TIGAR/cancer)
