# Supplementary material for: Deciphering the Controversial Role of TP53 Inducible Glycolysis and Apoptosis Regulator (TIGAR) in Cancer Metabolism as a Potential Therapeutic Strategy
Source: Cells. 2025 Apr 15;14(8):598. doi: 10.3390/cells14080598 (PMC12025843; doi:10.3390/cells14080598)
Supplement: Supplementary file 1 [file cells-14-00598-s001.zip › cells-3489250-supplementary.pdf]

Supplementary material

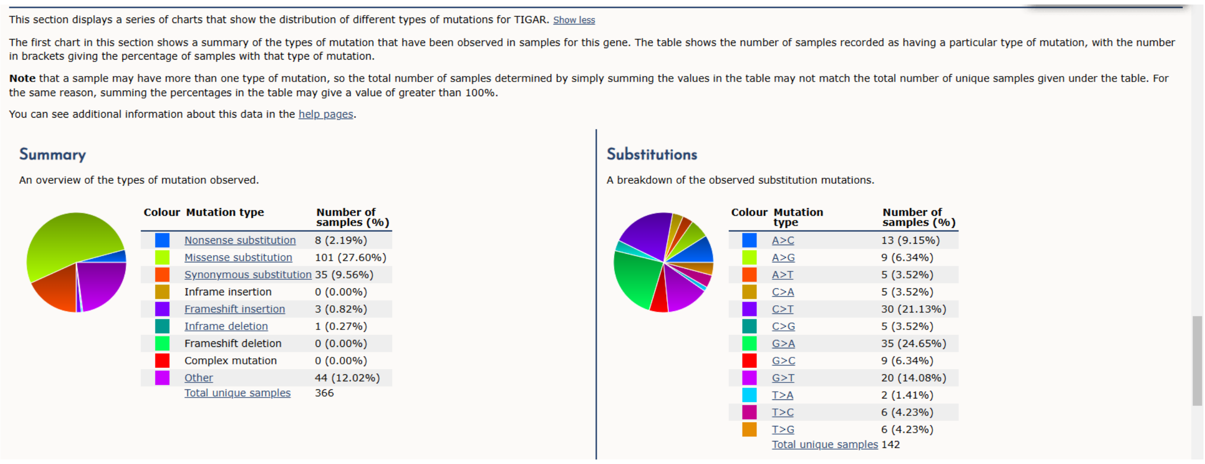

This section displays a series of charts that show the distribution of different types of mutations for TIGAR. Show less

The first chart in this section shows a summary of the types of mutation that have been observed in samples for this gene. The table shows the number of samples recorded as having a particular type of mutation, with the number in brackets giving the percentage of samples with that type of mutation.

Note that a sample may have more than one type of mutation, so the total number of samples determined by simply summing the values in the table may not match the total number of unique samples given under the table. For the same reason, summing the percentages in the table may give a value of greater than 100%.

You can see additional information about this data in the help pages.

Summary

An overview of the types of mutation observed.

Colour	Mutation type	Number of samples (%)
	Nonsense substitution	8 (2.19%)
	Missense substitution	101 (27.60%)
	Synonymous substitution	35 (9.56%)
	Inframe insertion	0 (0.00%)
	Frameshift insertion	3 (0.82%)
	Inframe deletion	1 (0.27%)
	Frameshift deletion	0 (0.00%)
	Complex mutation	0 (0.00%)
	Other	44 (12.02%)
	Total unique samples	366

Substitutions

A breakdown of the observed substitution mutations.

Colour	Mutation type	Number of samples (%)
	A>C	13 (9.15%)
	A>G	9 (6.34%)
	A>T	5 (3.52%)
	C>A	5 (3.52%)
	C>T	30 (21.13%)
	C>G	5 (3.52%)
	G>A	35 (24.65%)
	G>C	9 (6.34%)
	G>T	20 (14.08%)
	T>A	2 (1.41%)
	T>C	6 (4.23%)
	T>G	6 (4.23%)
	Total unique samples	142

Figure S1: TIGAR Single nucleotide polymorphisms (SNPs) identified on the COMIC website A. Summary of types of mutations observed in the TIGAR gene. B. Detailed representation of the substitution mutations observed in the TIGAR gene.

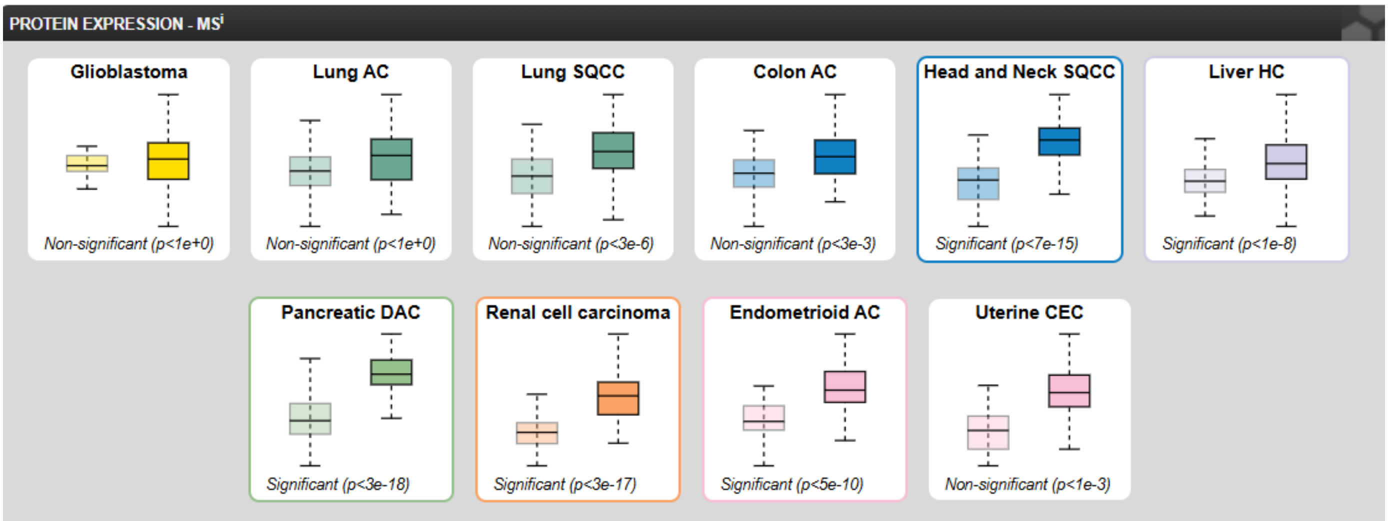

Figure S2: TIGAR expression in cancer versus normal tissues. nRPX is calculated as log2(intensity), derived from global protein abundance measurement at the protein level. These measurements are obtained through mass spectrometry performed by CPTAC on tissues using isobaric tandem mass tags (TMT). Retrieved through (https://www.proteinatlas.org/ENSG00000078237-TIGAR/cancer)
